# Sex-Specific Effects of Dietary Factors on Sarcopenic Obesity in Korean Elderly: A Nationwide Cross-Sectional Study

**DOI:** 10.3390/nu16081175

**Published:** 2024-04-15

**Authors:** Soojeong Kim, Kyung Hee Hong

**Affiliations:** 1 Department of Health Administration, Dongseo University, Busan 47011, Republic of Korea; sjkim@dongseo.ac.kr; 2 Department of Food Science and Nutrition, Dongseo University, Busan 47011, Republic of Korea

**Keywords:** dietary factor, diet quality, elderly, KNHANES, sarcopenic obesity

## Abstract

This study aimed to compare the dietary factors related to sarcopenia and obesity status in 5458 elderly individuals (2391 men and 3076 women) aged ≥65 years from the Korean National Health and Nutrition Examination Survey (2016–2019). Participants were categorized into normal, sarcopenia, obesity, and sarcopenic obesity groups. Sarcopenic obesity showed a higher prevalence of diabetes and lower HDL cholesterol levels compared to obesity. Sarcopenic obesity exhibited a lower total KHEI score and lower adequacy, including meat/fish/eggs/beans, than normal or obesity. In women, sarcopenic obesity scored lower than obesity on the total KHEI, adequacy for most foods, and balance of energy intake, and lower than sarcopenia on the adequacy of breakfast and milk/milk products. Sarcopenic obesity showed no significant difference in energy intake compared to sarcopenia, and less physical activity compared to sarcopenia and obesity, with a BMI/waist circumference comparable to that of obesity. Low total KHEI scores and scores for meat/fish/eggs/beans were most closely associated with sarcopenia in men and with sarcopenic obesity in women. In conclusion, low dietary quality and inadequate protein-rich foods are possibly associated with the prevalence of sarcopenic obesity in elderly Koreans, especially in women. Adequate energy intake and dietary diversity may be effective in managing sarcopenic obesity.

## 1. Introduction

With aging, body composition changes manifest as decreased physical activity and metabolic rate, resulting in a decline in muscle mass and strength, along with an increase in body fat, especially visceral abdominal fat. Consequently, alterations in body composition can lead to the onset of sarcopenia and central obesity [1]. Age-related sarcopenia and obesity can exert detrimental effects on chronic metabolic disorders and physical impairment, contributing to an increased risk of mortality [2]. Therefore, considering the global trend of a significant increase in the elderly population [3], sarcopenia and obesity in the elderly could pose a substantial threat to society, affecting not only individual well-being but also community health.

Sarcopenic obesity is a combination of sarcopenia and obesity, and the two conditions appear to be interconnected. Excessive fat mass associated with aging can induce sarcopenia. Obesity promotes muscle breakdown through insulin resistance, leading to a decline in both muscle mass and strength, consequently causing sarcopenia [4]. Conversely, a reduction in muscle mass and strength in sarcopenia leads to decreased energy expenditure, contributing to increased body fat and obesity induction [5]. The coexistence of sarcopenia and obesity in the elderly is associated with various health risks [6,7,8,9], and an increase in mortality rates [8], leading to undesirable long-term outcomes. Furthermore, sarcopenic obesity increases the vulnerability to health risks compared with sarcopenia or obesity alone. Sarcopenic obesity, compared to individuals with only muscle loss or obesity alone, is associated with a higher risk of mental disorders, an increased risk of hospitalization, elevated mortality rates, and a greater incidence of complications in various cancers [1]. Additionally, sarcopenic obesity has been found to increase the risk of metabolic diseases such as metabolic syndrome [10,11], cardiovascular disease [12], and diabetes [4], compared to either sarcopenia or obesity alone. Therefore, these findings underscore the importance of identifying and preventing the risk factors associated with sarcopenic obesity as a crucial strategy for promoting healthy aging.

Lifestyle modifications, including a healthy diet and adequate physical activity, have been elucidated as strategies to mitigate the risk of sarcopenic obesity [1,13]. Particularly, a high-quality diet plays a crucial role in both the risk and prevention of sarcopenic obesity. Overall, diet quality has been associated with a reduction in the risk of sarcopenic obesity in the elderly [14,15,16]. Additionally, there is an association between sarcopenic obesity and appropriateness of energy intake [17,18], energy ratio of macronutrients [18,19], and protein intake [20,21,22,23].

Based on data from the Korean National Health and Nutrition Examination Survey (KNHANES), the prevalence of sarcopenic obesity in elderly individuals aged 65 years and older has shown an increasing trend. In the years 2008–2011, the prevalence was 4.9% in men and 4.1% in women [19], while in 2016–2018, it increased to 6.6% in men and 16.2% in women [20]. Thus, age-related sarcopenic obesity is an emerging concern in South Korea. Although there is an association between sarcopenic obesity and dietary factors in Korean older adults, the majority of these studies were based on analyses of the 2008–2011 KNHANES [18,19,23,24,25]. Moreover, subsequent research on sarcopenic obesity in the elderly and its association with dietary factors, based on an analysis of the 2016–2018 KNHANES, has been limited to studies investigating regional differences in the association between dietary quality and sarcopenic obesity [14] and examining the nutritional intake of elderly men and women with sarcopenic obesity [20]. Therefore, it is necessary to examine dietary factors associated with age-related sarcopenic obesity using recent data. In this study, we aimed to investigate the dietary factors associated with sarcopenia, obesity, and sarcopenic obesity in elderly Korean men and women using the KNHANES of 2016–2019, the most recent data available on muscle strength, a diagnostic criterion for sarcopenia. Additionally, we analyzed the differences in dietary factors between sarcopenic obesity and sarcopenia or obesity.

## 2. Materials and Methods

### 2.1. Data Source and Study Population

This study utilized raw data from the KNHANES, which is representative of the general Korean population and widely used in national statistics. The study focused on participants aged 65 and above, including 6691 participants (2879 men and 3812 women) from the 6th (2016–2018) and 7th (2019) KNHANES. Excluding those who did not undergo grip strength and waist circumference measurements, participants without dietary survey data, and those who did not undergo blood tests, a total of 5458 elderly Korean participants (2391 men and 3067 women) were included in this study.

### 2.2. Definition of Sarcopenia and Obesity

In this study, sarcopenia was diagnosed based on muscle strength and handgrip strength (HGS) measured in the KNHANES. Recently, a definition of sarcopenia that considers muscle impairment in terms of muscle strength rather than mass has been proposed [1]. Sarcopenia and mortality exhibit a stronger relationship when diagnosed using qualitative measurements of muscles, such as HGS, rather than muscle mass [26]. HGS was measured using a digital grip strength dynamometer (TKK 5401 Grip-D; Takei, Tokyo, Japan). The maximum value of the dominant hand, which was primarily used for three measurements (2016–2018), or the maximum value measured in 2019, was used. According to the Asian Working Group for Sarcopenia (AWGS) 2019 criteria [27], individuals with HGS < 28 kg for men and <18 kg for women were diagnosed with low HGS and classified as having sarcopenia. Obesity was diagnosed based on waist circumference, with the Korean Society for the Study of Obesity (KSSO) cutoffs defining abdominal obesity as ≥90 cm for men and ≥85 cm for women [28]. Sarcopenic obesity was defined as the coexistence of low HGS and abdominal obesity, whereas non-sarcopenic and non-abdominal obese individuals were classified as normal.

### 2.3. Health and Dietary Behavioral Characteristics

Health behavioral variables, such as smoking, alcohol consumption, perceived stress, number of days per week walking for 10 min or more, and number of days per week walking for 30 min or more, were identified. Alcohol consumption was defined as having consumed alcohol at least once a month in the past year, whereas perceived stress was defined as feeling a significant amount of stress in daily life. Dietary behavioral variables, including nutrition education experience, frequency of eating out, food security, and use of dietary supplements, were investigated. The energy intake distribution (%) for each meal (breakfast, lunch, dinner, and snacks) was analyzed. Nutritional education was defined as having received nutritional education or counseling within the past year. Food security was determined based on whether all family members had enough of the desired quantity and variety of food in the previous year. Dietary supplement intake was defined as regular consumption of dietary supplements for at least two weeks within the past year.

### 2.4. Diet Quality (KHEI)

Diet quality was assessed using the Korean Healthy Eating Index (KHEI) developed by the Korean Centers for Disease Control and Prevention (KCDC). It comprises 14 components based on healthy eating habits among Koreans. Specifically, it includes eight items for the adequacy of daily intake needed for a quality diet (having breakfast, intake of mixed grains, fruits, fresh fruits, vegetables, vegetables excluding kimchi and pickles, meat/fish/eggs/beans, and milk and milk products), three items for moderation that should be limited (percentage of energy intake from saturated fatty acids, sweets/beverages, and sodium), and three items for balancing energy intake (percentage of energy intake from carbohydrate, fat, and adequate energy intake). Each item was scored on a scale of 5–10 points, resulting in a total of 100 points (Supplementary Table S1).

### 2.5. Nutrient Intake

Variables related to nutrient intake were assessed based on dietary intake survey data collected using the 24 h recall method conducted in the KNHANES. Energy, macronutrients (carbohydrates, proteins, and fats), dietary fiber, minerals (calcium, phosphorus, sodium, potassium, and iron), and vitamins (vitamin A, thiamine, riboflavin, niacin, and vitamin C) were analyzed. Additionally, the percentages of energy intake from carbohydrates, proteins, and fats were examined.

### 2.6. Statistical Analyses

The raw data of the KNHANES were analyzed considering the complex sample design information (strata, clusters, and weights) due to multistage clustered sampling. All analyses were performed using the SPSS Statistics ver. 28.0 (IBM SPSS, Armonk, NY, USA). Complex sample cross-tabulation and complex sample general linear model procedures were used to compare the distribution of general characteristics, biochemical measures, health and dietary behaviors, KHEI, and nutrient intake among different groups of study participants; complex sample cross-tabulation and Complex Samples General Linear Model (CSGLM) procedure were utilized. The results of the complex sample cross-tabulation are presented as the unweighted frequency and population estimated percentage.

Group differences were assessed using Pearson’s chi-squared test. The results of the CSGLM analysis were presented as estimated means and standard errors, and group differences in means were assessed using Wald’s F statistics. The sarcopenic obesity group was used as the reference group, and the LSD method was used to test mean differences. Additionally, to identify independent factors affecting sarcopenia, obesity, and sarcopenic obesity compared with the normal group, we conducted complex sample logistic regression analysis and calculated odds ratios (OR) and 95% confidence intervals (CI). In this analysis, three models were employed: model 1 was crude; model 2 was adjusted for age; model 3 was adjusted for education, household income, and physical activity for more than 30 min (days/week), and for women, adjusted for age, education, region, household income, and physical activity for more than 30 min (days/week). Statistical significance was set at *p* < 0.05.

## 3. Results

### 3.1. General Characteristics

This study included 2391 men and 3067 women. Among men, 1010 (44.2%) were in the normal group, 466 (19.9%) had sarcopenia, 709 (27.8%) were obesity, and 206 (8.0%) had sarcopenic obesity. There were 992 (32.7%) women in the control group: 588 (19.6%) with sarcopenia, 930 (29.7%) with obesity, and 557 (18.0%) with sarcopenic obesity. The general characteristics of the participants are presented in Table 1. Education level, employment rate, and urban residency were lower in both men and women in the sarcopenia and sarcopenic obesity groups compared to the normal and obesity groups. Among the women, the sarcopenic obesity group had lower education and employment rates than the sarcopenia group. Income levels were also lower in the sarcopenia and sarcopenic obesity groups in both sexes, with the sarcopenic obesity group exhibiting the lowest income level. Specifically, a high percentage of participants in the sarcopenic obesity group reported low and middle–low household incomes (86.7% of men and 82.6% of women). The prevalence of diabetes was the highest in the sarcopenic obesity group, with rates of 32.8% in men and 31.4% in women, surpassing the obesity and sarcopenia groups in both sexes. The prevalence of osteoporosis was highest in women, followed by sarcopenia, sarcopenic obesity, and normal weight and obesity groups. Among men, the sarcopenic obesity group had a significantly lower BMI than the obesity group, whereas among women, there was no significant difference in BMI between the sarcopenic obesity and obesity groups.

### 3.2. Clinical Characteristics

Table 2 presents differences in clinical characteristics with sarcopenic obesity as the reference. The sarcopenic obesity group exhibited significantly higher levels of fasting glucose, HbA_1_c, insulin, HOMA-IR, and triglycerides compared to the normal group, with no significant differences from the obesity group in both sexes. Compared to the sarcopenia group, men in the sarcopenic obesity group had higher HbA_1_c, insulin, and HOMA-IR levels, while women had higher fasting glucose, HbA_1_c, and insulin levels. Plasma HDL-cholesterol concentration was the lowest in the sarcopenic obesity group. Plasma total cholesterol concentration was lower in the sarcopenic obesity group than the normal group in women, with no significant differences among groups in men. Plasma LDL-cholesterol concentration showed no significant differences among groups in both sexes.

### 3.3. Health and Dietary Behavioral Characteristics

Table 3 presents the health- and diet-related behavioral characteristics of the participants. In the sarcopenia and sarcopenic obesity groups, lower rates of alcohol consumption and eating out, along with higher levels of perceived stress, were observed in both sexes. In men, the sarcopenia group tended to have a lower frequency of eating out than the sarcopenic obesity group. There was no significant difference in the proportion of food insecurity among the groups for men, whereas for women, the sarcopenic obesity group had the highest proportion (17.1%). The proportion of individuals taking dietary supplements was higher in the normal group, with 79.0% of men and 61.0% of women, surpassing other groups in both sexes; among women, the sarcopenic obesity group had the lowest rate of dietary supplement intake. In terms of physical activity, when comparing the number of days with activity for ≥10 min and ≥30 min per week, the sarcopenic obesity group was significantly lower than the normal group in men (*p* < 0.05, *p* < 0.01, respectively). In women, the sarcopenic obesity group had significantly lower levels of physical activity for ≥10 min compared to the normal group, and even lower than the obesity group (all, *p* < 0.001). Furthermore, for physical activity for ≥30 min, the sarcopenic obesity group showed significantly lower levels compared to the sarcopenia group (*p* < 0.01). Women with sarcopenia, with or without obesity, showed a significantly lower proportion of energy intake from snacks and a higher proportion of energy intake during breakfast and dinner, indicating a higher proportion of energy intake from regular meals. However, no such group differences were observed among men. Smoking status and experience with nutrition education showed no significant differences between the groups for either sex.

### 3.4. Diet Quality (KHEI)

Differences in the KHEI scores among the groups were compared with the sarcopenic obesity group as a reference, and the results are presented in Table 4. The total KHEI score of the sarcopenic obesity group was lower than that of the normal group in both men and women (*p* < 0.01, *p* < 0.001, respectively). Additionally, in women, the total KHEI score of the sarcopenic obesity group was lower than that of the obesity group (*p* < 0.001). Among men, the sarcopenic obesity group exhibited lower scores in components including the intake of total vegetables, vegetables excluding kimchi and pickles, meat/fish/eggs/beans, and % energy from fat compared to both the normal and obesity groups. In addition, the sarcopenic obesity group had a lower total fruit intake score than the normal group. Moreover, the sarcopenic obesity group had higher scores for mixed grain intake and meat/fish/egg/bean intake than the sarcopenia group. In contrast, the sodium intake score in men with sarcopenic obesity was significantly higher than those in normal and obese men. Meanwhile, in women, additional component scores, including total fruit intake, % energy from carbohydrates, and energy intake, were lower in the sarcopenic obesity group compared to the normal and obesity groups, resulting in most KHEI component scores of the sarcopenic obesity group being lower than those of the normal and obesity groups. Furthermore, sarcopenic obese women had lower scores for having breakfast, not only compared to the normal group but also compared to sarcopenic women (*p* < 0.01, *p* < 0.05, respectively). The scores for milk and milk product intake were the lowest among the four groups and were significantly lower than those of the other three groups (all *p* < 0.001). On the other hand, the % energy from sweet and beverage component scores was higher not only in the obesity group but also in the normal group.

### 3.5. Nutrient Intake

We compared the daily nutrient intake adjusted for age and total energy intake among the groups using the sarcopenic obesity group as a reference (Table 5). In both men and women, total energy intake was significantly lower in the sarcopenic obesity group than in the normal (*p* < 0.01 men; *p* < 0.05) and obesity groups (all, *p* < 0.05). Among men, dietary fiber intake (*p* < 0.001 vs. normal; *p* < 0.01 vs. Obesity) and potassium intake (all *p* < 0.05) were significantly lower in the sarcopenic obesity group than in the normal and obesity groups. In women, potassium intake was lower in the sarcopenic obesity group than in the obesity group (*p* < 0.05), whereas sodium intake was significantly higher than that in the normal group (*p* < 0.05). Additionally, sarcopenic obese women had lower vitamin C intake than both the normal (*p* < 0.01) and obesity groups (*p* < 0.05). In women, the percentage of energy derived from proteins was higher in the sarcopenic obesity group than in the sarcopenia group.

### 3.6. Association of KHEI with Sarcopenia and Obesity Status

The ORs for sarcopenia, obesity, and sarcopenic obesity according to the total KHEI score and the meat/fish/eggs/bean intake components are presented in Table 6. In men, the ORs for sarcopenia (OR: 3.46, 95% CI: 2.27–5.26), obesity (OR: 1.62, 95% CI: 1.13–2.32), and sarcopenic obesity (OR: 2.00, 95% CI: 1.22–3.27) were highest in the Q1 of the total KHEI score. After adjusting for age, the prevalence remained elevated in Q1 for sarcopenia (OR: 3.02, 95% CI: 1.96–4.66), obesity (OR: 1.61, 95% CI: 1.12–2.30), and sarcopenic obesity (OR: 1.75, 95% CI: 1.05–2.91). Even after adjusting for confounding factors, sarcopenia remained associated with KHEI scores in Q1 (OR: 2.76, 95% CI: 1.67–4.58) and Q2 (OR: 2.06, 95% CI: 1.30–3.28). Additionally, obesity showed an association with KHEI score Q1 (OR: 1.70, 95% CI: 1.14–2.56). In women, the association with the total KHEI score was observed for sarcopenia and sarcopenic obesity, with the highest prevalence in the Q1 for both sarcopenia (OR: 2.31, 95% CI: 1.60–3.34) and sarcopenic obesity (OR: 2.92, 95% CI: 2.01–4.23). After adjusting for age, only sarcopenic obesity remained associated with KHEI scores in Q1 (OR: 1.70, 95% CI: 1.13–2.54) and Q2 (OR: 1.52, 95% CI: 1.05–2.20).

An association between the meat/fish/eggs/bean intake component score and both sarcopenia and sarcopenic obesity was observed in both sexes. The prevalence of sarcopenia (OR: 2.52, 95% CI: 1.75–3.64 for men; OR: 2.76, 95% CI: 1.96–3.88 for women) was highest in Q1 for both sexes. Additionally, the prevalence of sarcopenic obesity was highest in men in Q2 (OR: 2.07, 95% CI: 1.26–3.40) and in women in Q1 (OR: 2.36, 95% CI: 1.62–3.45). After adjusting for age, the odds of sarcopenia and sarcopenic obesity were higher in men in Q2 (OR: 2.09, 95% CI: 1.42–3.08 for sarcopenia; OR: 1.78, 95% CI: 1.06–2.98 for sarcopenic obesity), and in women, the odds were higher in Q1 (OR: 1.63, 95% CI: 1.15–2.32 for sarcopenia; OR: 1.72, 95% CI: 1.14–2.58 for sarcopenic obesity). After adjusting for confounding factors, sarcopenia remained associated with meat/fish/eggs/beans intake in Q1 (OR: 1.77, 95% CI: 1.13–2.87) and Q2 (OR: 1.81, 95% CI: 1.14–2.87) in men, and Q3 (OR: 1.58, 95% CI: 1.01–2.48) in women.

## 4. Discussion

This study compared dietary factors related to sarcopenia, obesity, and sarcopenic obesity among adults aged 65 and older who participated in the KNHANES from 2016 to 2019. We found that among elderly women, the proportion of the normal group was lower than that of elderly men, whereas the proportion of sarcopenic obesity was higher. Participants diagnosed with sarcopenia, with or without obesity, exhibited lower levels of education and employment and were more likely to reside in rural areas. Additionally, their income levels were lower, with those of sarcopenic obesity being even lower than those of sarcopenia. Therefore, the socioeconomic status of older adults is lower in those with sarcopenic obesity than in those with sarcopenia or obesity alone.

In this study, fasting blood glucose, HbA_1_c, insulin, and insulin resistance levels were elevated in the sarcopenic obesity group compared to those in the obesity group, with no significant differences. However, the prevalence of diabetes in the sarcopenic obesity group was even higher, reaching 32.8% in men and 31.4% in women, surpassing the rates observed in the obesity group for both sexes, despite their BMI values being lower than or comparable to those of the obesity group. In addition, the sarcopenic obesity group exhibited the lowest plasma HDL-cholesterol concentration in both sexes. Type 2 diabetes associated with sarcopenic obesity is highly prevalent in adults in the United States [6], and sarcopenic obesity was associated with a 2.16-fold increase in the odds of diabetes in elderly Koreans [7]. Consistent with our findings, in the KNHANES study conducted from 2008 to 2011, elderly individuals with sarcopenic obesity had a diabetes prevalence of 24.2%, surpassing the 18.5% observed in those with obesity [19]. However, we found that there was an even higher increase in the prevalence of diabetes than that in our study. Fasting blood glucose was found to be highly associated with sarcopenic obesity in elderly Koreans aged 75–84 [29]. Our study results align with previous findings indicating elevated fasting blood glucose [24,25] and insulin levels, along with increased HOMA-IR [25], in obese elderly individuals with or without sarcopenia. Furthermore, in line with our study findings, prior research on the Korean elderly reported that HDL-cholesterol in elderly individuals with sarcopenic obesity was the lowest among the normal, sarcopenia, and sarcopenic obesity groups in both men [17] and women [25]. Additionally, in postmenopausal Korean women, sarcopenic obesity has been reported to pose a higher risk of metabolic syndrome than obesity alone [11]. Therefore, sarcopenic obesity is considered more vulnerable to the risk of metabolic syndromes, including diabetes, than sarcopenia or obesity alone.

In our study, both men and women exhibited lower alcohol consumption and eating-out frequency, along with higher stress perception. Among women, the sarcopenic obesity group had the lowest prevalence of dietary supplement intake and the highest food insecurity compared to the normal, sarcopenia, and obesity groups. However, such differences were not observed in men, indicating that elderly women with sarcopenic obesity exhibited the worst dietary characteristics, making them particularly vulnerable to healthy diets. Food insecurity increases the likelihood of obesity and sarcopenia in the elderly [4]. Therefore, improving food insecurity could be a strategy for reducing the risk of sarcopenic obesity. Additionally, the impact of food insecurity on obesity status differs by sex, as there was no association between food insecurity and obesity in elderly men [4]. Consequently, food insecurity is thought to have a more detrimental effect on women than on men among the elderly.

Furthermore, sex-based differences in physical activity were evident in our findings related to sarcopenic obesity. Elderly women with sarcopenic obesity exhibited the lowest level of physical activity compared to the normal and sarcopenia and obesity groups. In contrast, among older men, there was no significant difference in physical activity between those with sarcopenic obesity and those with sarcopenia or obesity. Similar to our findings, a previous study found no significant difference in regular exercise rates between elderly men with and without sarcopenic obesity; however, in women, those with sarcopenic obesity exhibited a lower rate of regular exercise [18] and decreased levels of various physical activities compared to those without sarcopenia [30]. Consequently, insufficient physical activity among elderly women has been highlighted as a significant concern, suggesting that a lack of physical activity in women as they age could be a major risk factor for sarcopenic obesity. In the elderly, an imbalance between energy intake and expenditure, often associated with a sedentary lifestyle or lack of physical activity, is linked to the maintenance of or increase in skeletal muscle mass [31]. Consistent with our study findings, Koreans aged 40 and older demonstrated the lowest prevalence of sarcopenic obesity among those engaging in sufficient physical activity, compared with both sarcopenia and obesity [14]. Insufficient physical activity in the Korean elderly was associated with threefold higher odds of sarcopenic obesity [19], and as physical activity levels increased, the OR for sarcopenic obesity decreased, reaching 0.55 for men and 0.43 for women in the highest activity group [17]. Moreover, increased physical activity in elderly Canadian individuals is associated with higher lean mass and lower fat mass [15], and Germans aged 50 years and above with moderate weekly activity had lower odds of low handgrip strength [22]. Notably, resistance training has been reported as an intervention method for improving muscle function in individuals with sarcopenic obesity [32]. In adults with sarcopenic obesity, resistance exercise reduces fat mass and improves grip strength, and the combination of aerobic and resistance exercises leads to decreased fat mass and improved walking speed [33]. Therefore, exercise, particularly resistance exercise, is crucial for the prevention and improvement of sarcopenic obesity. Taken together, this study demonstrates sex differences in health- and dietary-related behaviors among the elderly with sarcopenic obesity, with women exhibiting the poorest behaviors, even when compared to those with sarcopenia or obesity alone. This suggests that older women with sarcopenic obesity may be less attentive to their health, making them more vulnerable to health concerns.

Overall, we observed that the dietary quality in elderly individuals with sarcopenia, with or without obesity, was lower than that in those with normal or obesity in both men and women. Women with sarcopenic obesity exhibited notably lower diet quality, showing significantly lower overall dietary quality (total KHEI score) than the obesity group, whereas in men, no significant difference was observed in the KHEI scores between sarcopenic obesity and sarcopenia or obesity. Furthermore, men with sarcopenic obesity had lower scores on the adequacy of vegetables, protein sources, and balance of fat intake. For women, not only were these scores lower, but their scores for adequacy of fruit, milk, and dairy products, and balance of carbohydrate and energy intake, were also lower than those in the obesity group. Consequently, the healthy eating index for sarcopenic obesity is generally less favorable than that for obesity, especially in women. Even more notably, in women, sarcopenic obesity showed significantly lower adequacy of breakfast and milk/milk products compared to sarcopenia, whereas men with sarcopenic obesity exhibited no fewer healthy eating index components than those with sarcopenia. Therefore, in elderly individuals with sarcopenic obesity, women exhibit a more pronounced deficiency in the intake of breakfast, fruits, and milk products than men. Overall, the dietary quality of patients with sarcopenic obesity appears to be more compromised in women than in men. Similar to our study findings, an analysis of KNHANES data for individuals aged 40 and above found lower KHEI scores in those with sarcopenia and sarcopenic obesity compared to normal and obesity groups, especially in urban residents [14]. Moreover, a study of Canadian elderly found that lower nutritional risk was associated with higher lean mass and lower fat mass [15], and a healthy dietary score was associated with sarcopenic obesity in Spanish elderly [16]. Thus, these results indicate that overall dietary quality is associated with sarcopenic obesity. In our study, the comprehensive poor quality of diet, including poor food diversity and imbalance in energy intake, was identified as a risk factor for sarcopenic obesity, particularly among elderly women.

In our study, the sarcopenia group showed a significantly lower energy intake than the obesity group; however, their BMI and waist circumference were comparable. Ultimately, the sarcopenic group exhibited obesity levels akin to those of the obesity group but had energy intake similar to that of the sarcopenia group, highlighting insufficient energy intake as a crucial risk factor for sarcopenic obesity. Elderly women with sarcopenic obesity have a notably lower proportion of their energy intake from snacks. This suggests an overall concern regarding energy intake, given the low total energy from regular meals and limited energy obtained from snacks. Consistent with our findings, previous studies revealed that sarcopenic obese individuals showed lower energy intake than the obese group in Korean elderly [24] and American children and adolescents [34]. Moreover, in the Korean elderly, insufficient energy intake has been reported as a risk factor for sarcopenic obesity, with an OR of 1.25 when energy intake was below the recommended level [17], and an inverse association was observed, with an OR of 0.95 for sarcopenic obesity per 100 calorie increment in total calorie intake [18]. Additionally, maintaining energy intake above the estimated energy requirement while meeting the recommended physical activity levels significantly reduced the likelihood of sarcopenic obesity, with an OR of 0.32 [19]. A meta-analysis also confirmed that energy restriction in the elderly leads to muscle loss. Furthermore, even with a concurrent high protein intake, insufficient energy intake has been shown to increase the risk of sarcopenic obesity in the elderly [13]. Similarly, a meta-analysis indicated that a low-calorie, high-protein diet did not contribute to an increase in muscle mass or strength [33]. Hypocaloric high-protein diets do not appear to be an effective strategy for treating sarcopenic obesity [35]. Therefore, diets that solely restrict energy intake have emerged as significant risk factors for age-related sarcopenic obesity, highlighting the crucial importance of adequate energy intake in the prevention and management of sarcopenic obesity in the elderly.

The study results also revealed sex differences in nutrient intake. Among men with sarcopenic obesity, fiber and potassium intake were lower than those in the normal and obesity groups, likely due to insufficient vegetable consumption. Additionally, women with sarcopenic obesity exhibited higher sodium but lower potassium and vitamin C intakes, suggesting that their simplistic diet, characterized by insufficient consumption of diverse foods such as vegetables, fruits, meat/fish, and dairy products, may be the cause. Similar observations have been reported, with vitamin C insufficiency negatively associated with sarcopenic obesity in Spanish elderly [16], and the OR for sarcopenic obesity increased to 1.3 when vitamin C intake was less than the recommended intake in Korean elderly [17]. These results confirmed that a lack of diversity in foods, especially those rich in vitamin C, such as fruits and vegetables, contributed to an elevated risk of sarcopenic obesity. The study found that both men and women surpassed the recommended range of the KDRI (55–65%) in energy proportion from carbohydrates, particularly women, who exhibited a higher range of 71.6–73.2%. Additionally, in line with this, the carbohydrate energy balance score of the KHEI was low for both sexes, ranging from 1.3 to 2.0 out of a possible 5 points. Notably, women with sarcopenic obesity had lower carbohydrate energy balance scores than those in the obesity group. Song et al. reported that in middle-aged Koreans at high risk for metabolic disease, a higher proportion of energy intake from carbohydrates was associated with a lower intake of meat, fish, vegetables, fruits, milk, and dairy products, and a higher intake of kimchi, pointing to the issue of a high-carbohydrate, less diverse diet [36]. These results suggest that an insufficient total energy intake and a monotonous dietary pattern with limited food variety, resulting in a predominant reliance on carbohydrates for the majority of energy, may increase the risk of sarcopenic obesity.

Imbalances in macronutrient intake appear to affect body composition (that is, body fat and muscle mass), and thus contribute to the development of sarcopenic obesity. Our study findings align with this, showing consistently high carbohydrate energy intake ratios exceeding 75% across all four groups of elderly Koreans, regardless of sarcopenia and obesity status [19]. Previous studies have shown that overconsumption of carbohydrate-rich foods such as wheat led to a 2.1-fold increase in the prevalence of sarcopenic obesity [21], and an inverse relationship was also reported between carbohydrate intake (g/kg body weight) and sarcopenic obesity [18], suggesting that carbohydrate intake could be the most relevant index for determining sarcopenic obesity in the Korean elderly. Additionally, elderly women with sarcopenic obesity have a higher percentage of energy from fat than those without sarcopenic obesity [18]. In contrast, in U.S. children and adolescents with a low-carbohydrate energy ratio (51.0–52.3%) and a high-fat energy ratio (33.5–34.4%), a diet low in carbohydrates and high in fat was associated with an increased risk of sarcopenic obesity [34].

Protein intake is also associated with sarcopenic obesity. In the Korean elderly, sarcopenic obese individuals were found to have a lower nutrient adequacy ratio of proteins than non-sarcopenic obese individuals [20]. Moreover, in elderly rural women, the consumption of protein-rich foods, such as meat, is inversely associated with the prevalence of sarcopenic obesity [21]. Similar associations were observed in Europeans aged 50–70, where the daily intake of meat and fish was associated with lower odds of sarcopenic obesity [22]. High protein intake during weight loss helps prevent muscle loss, especially when combined with exercise [35]. Intervention studies have shown that concurrent high protein intake (1.2–1.4 g/kg body weight) in sarcopenic obesity reduces the risk of muscle loss due to reduced energy intake, as demonstrated in Italian women aged 41–76 [37]. Meta-analyses have suggested that the most effective strategy for addressing sarcopenic obesity in elderly individuals involves a combination of appropriate exercise and a high-protein diet [13]. Another meta-analysis highlighted the importance of a high-protein diet for managing sarcopenic obesity in adults, emphasizing the need for fat loss and muscle mass retention [33]. Taken together, our findings suggest imbalanced macronutrient ratios, excessive carbohydrate levels, and insufficient total protein adequacy in elderly participants, particularly women. We believe that ensuring adequate energy and protein intake is crucial for the prevention and management of sarcopenic obesity.

In our study, the association between diet quality, as measured by the total KHEI score, and sarcopenia, obesity, and sarcopenic obesity, revealed sex differences. In men, lower diet quality, as represented by the age-adjusted ORs in Q1, increased the risk of sarcopenia (3.02), sarcopenic obesity (1.75), and obesity (1.61), with sarcopenia exhibiting the most substantial increase in risk. Among women, a lower diet quality in Q1 increased the ORs for sarcopenic obesity (2.92) and sarcopenia (2.31). Examination of the adequacy of protein sources in the diet, specifically meat/fish/eggs/bean intake, in relation to sarcopenia, obesity, and sarcopenic obesity, revealed that inadequate protein source adequacy increased the odds of both sarcopenia and sarcopenic obesity in both sexes. In men, the age-adjusted ORs in Q2 showed that sarcopenia (2.09) was higher than sarcopenic obesity (1.78), whereas in women, Q1 had a higher OR for sarcopenic obesity (1.72) than for sarcopenia (1.63). Collectively, our findings indicate that diet quality has a sex-specific effect on sarcopenia and obesity. Lower overall diet quality and inadequate protein intake were strongly associated with an increased risk of sarcopenia in men and sarcopenic obesity in women.

This study had some limitations. First, as it was an analysis of KNHANES data with a cross-sectional design, causal relationships could not be determined. Second, dietary data were obtained using the 24 h recall method, which may not fully reflect the participants’ usual dietary habits. Third, the diverse definitions of muscle loss contribute to the lack of a consensus on the definition of sarcopenia. Notably, KNHANES 2016–2019 did not measure muscle mass. Therefore, the weakness of this study lies in its inability to evaluate muscle mass because it utilized grip strength as a criterion for diagnosing sarcopenia. Nevertheless, the strength of this study is that it comprehensively analyzed the association of health behaviors, dietary quality, and nutrient intake with sarcopenia and obesity status in elderly Korean men and women based on national big data.

## 5. Conclusions

The findings of this study indicate that concurrent low energy intake and expenditure play a significant role in the development of sarcopenic obesity, and that adequate and balanced diets, rather than simply focusing on energy restriction, may contribute to the prevention of sarcopenia and obesity in the elderly. Moreover, poor diet quality exerts sex-specific effects, with a stronger association between sarcopenia in elderly men and sarcopenic obesity in elderly women. In particular, elderly women with lower levels of education and income demonstrated evident vulnerability. Their poor dietary quality, characterized by low food diversity, led to the development of sarcopenic obesity, thereby increasing their susceptibility to diabetes compared to individuals with either sarcopenia or obesity alone. Therefore, a prompt diagnosis and proactive intervention are crucial for managing sarcopenic obesity. A healthy dietary pattern is a strategic approach for reducing the risk of age-related sarcopenic obesity, contributing to healthy aging. The results of this study provide valuable insights for planning dietary interventions to address sarcopenic obesity in the elderly Korean population. To advance the prevention, treatment, and combat against sarcopenic obesity, further research using diverse approaches is required. Therefore, clinical trials involving various interventions should be conducted to identify the most effective strategies. Therefore, studies investigating the association between protein intake levels, combinations of physical activity and diet, and the risk of sarcopenia and obesity in elderly individuals, especially those with diabetes, are needed.

## Figures and Tables

**Table 1:** General characteristics of subjects.

Variables	Men (*n* = 2391)					Women (*n* = 3067)				
	Normal	Sarcopenia	Obesity	Sarcopenic Obesity	*p*-Value ^2^	Normal	Sarcopenia	Obesity	Sarcopenic Obesity	*p*-Value
Total	1010 (44.2) ^1^	466 (19.9)	709 (27.8)	206 (8.0)		992 (32.7)	588 (19.6)	930 (29.7)	557 (18.0)	
Age (y)	71.4 ± 0.2 ^3^ ***	75.9 ± 0.3	71.9 ± 0.2 ***	75.4 ± 0.4	<0.001	71.0 ± 0.2 ***	75.4 ± 0.2	71.8 ± 0.2 ***	75.3 ± 0.2	<0.001
Hand grip strength (kg)	35.3 ± 0.2 ***	22.9 ± 0.2	36.0 ± 0.2 ***	22.9 ± 0.4	<0.001	22.1 ± 0.1 ***	14.1 ± 0.1	22.6 ± 0.1 ***	14.3 ± 0.2	<0.001
Waist circumference (cm)	82.4 ± 0.2 ***	81.1 ± 0.4 ***	95.9 ± 0.2	95.6 ± 0.3	<0.001	78.0 ± 0.2 ***	76.9 ± 0.3 ***	91.7 ± 0.3	91.8 ± 0.3	<0.001
Body mass index (kg/m^2^)	22.5 ± 0.1 ***	21.6 ± 0.1 ***	26.4 ± 0.1 *	25.9 ± 0.2	<0.001	22.6 ± 0.1 ***	21.9 ± 0.1 ***	26.7 ± 0.1	26.8 ± 0.1	<0.001
Marital status										
Married	1001 (99.2)	463 (99.4)	706 (99.7)	204 (99.3)	0.621	987 (99.3)	587 (99.7)	921 (99.3)	550 (98.8)	0.511
Single	9 (0.8)	3 (0.6)	3 (0.3)	2 (0.7)		5 (0.7)	1 (0.3)	9 (0.7)	7 (1.2)	
Education										
≤Elementary school	339 (34.0)	244 (53.1)	221 (31.6)	101 (49.9)	<0.001	568 (57.9)	423 (77.2)	634 (67.4)	433 (81.5)	<0.001
Middle school	181 (18.1)	56 (14.3)	129 (19.9)	42 (24.6)		149 (15.1)	46 (8.5)	130 (14.5)	55 (10.8)	
High school	264 (26.9)	76 (18.9)	197 (27.9)	33 (19.1)		158 (18.3)	40 (8.9)	98 (13.5)	22 (5.1)	
≥College	192 (21.0)	42 (13.6)	123 (20.6)	12 (6.5)		71 (8.7)	25 (5.5)	31 (4.5)	12 (2.6)	
Employment status										
Employed	436 (43.4)	142 (32.6)	278 (38.7)	62 (31.4)	0.004	281 (28.3)	119 (19.6)	290 (28.9)	104 (18.7)	<0.001
Unemployed	538 (56.6)	277 (67.4)	393 (61.3)	126 (68.6)		666 (71.7)	415 (80.4)	603 (71.7)	418 (81.3)	
Region										
City	754 (79.8)	320 (76.7)	521 (80.2)	128 (72.1)	0.134	752 (83.2)	372 (71.8)	687 (80.8)	382 (74.8)	<0.001
Rural area	256 (20.2)	146 (23.3)	188 (19.8)	78 (27.9)		240 (16.8)	216 (28.2)	243 (19.2)	175 (25.2)	
Household income										
Low	341 (32.7)	268 (54.5)	255 (34.1)	132 (62.1)	<0.001	429 (40.4)	359 (58.4)	481 (50.0)	344 (59.8)	<0.001
Middle-low	350 (33.1)	107 (23.3)	217 (29.6)	51 (24.6)		298 (29.0)	111 (20.3)	249 (24.0)	120 (22.8)	
Middle-high	175 (18.4)	62 (15.2)	141 (20.9)	15 (7.7)		162 (17.9)	73 (13.1)	122 (14.7)	48 (9.6)	
High	139 (15.7)	23 (7.1)	92 (15.3)	8 (5.7)		100 (12.7)	40 (8.2)	75 (11.3)	42 (7.8)	
Diseases										
Diabetes Mellitus	181 (16.9)	99 (20.8)	202 (28.8)	69 (32.8)	<0.001	139 (13.5)	116 (20.1)	231 (23.0)	177 (31.4)	<0.001
Osteoporosis	26 (2.7)	29 (5.4)	20 (3.3)	8 (3.3)	0.267	378 (38.8)	260 (44.4)	320 (34.0)	205 (37.8)	0.014
Depression	27 (2.5)	19 (4.8)	17 (2.0)	13 (6.4)	0.020	87 (9.3)	53 (11.0)	71 (8.5)	59 (12.1)	0.303

^1^ *n* (weighted %). ^2^ *p*-value by chi test for categorical variables and general linear regression for continuous variables. ^3^ Mean ± standard error. * *p* < 0.05, *** *p* < 0.001 versus sarcopenic obesity by LSD multiple comparisons.

**Table 2:** Clinical characteristics according to sarcopenia and obesity status by sex.

Variables	Men					Women				
	Normal	Sarcopenia	Obesity	Sarcopenic Obesity	*p*-Value ^2^	Normal	Sarcopenia	Obesity	Sarcopenic Obesity	*p*-Value
Fasting glucose (mg/dL)	106.5 ± 1.0 ^1,^*	107.5 ± 1.6	114.3 ± 1.4	113.9 ± 2.9	<0.001	101.1 ± 0.7 ***	104.1 ± 1.4 ***	110.9 ± 1.4	111.4 ± 1.2	<0.001
HbA_1_c (%)	5.9 ± 0.1 ***	5.9 ± 0.1 ***	6.2 ± 0.1	6.3 ± 0.1	<0.001	5.9 ± 0.02 ***	5.9 ± 0.04 ***	6.2 ± 0.1	6.3 ± 0.1	<0.001
Insulin (uIU/mL)	6.8 ± 0.4 **	6.6 ± 0.5 **	12.1 ± 0.8	12.0 ± 1.8	<0.001	7.0 ± 0.3 ***	8.7 ± 1.0 *	11.9 ± 0.5	11.9 ± 1.0	<0.001
HOMA-IR	1.8 ± 0.1 **	1.7 ± 0.1 **	3.7 ± 0.4	3.4 ± 0.6	<0.001	1.90 ± 0.14 ***	2.5 ± 0.4	3.3 ± 0.2	3.5 ± 0.4	<0.001
Systolic BP (mm Hg)	125.9 ± 0.7	126.3 ± 1.0	127.9 ± 0.7	126.5 ± 1.5	0.206	129.1 ± 0.7	130.6 ± 0.9	131.1 ± 0.9	130.8 ± 1.0	0.221
Diastolic BP (mm Hg)	72.9 ± 0.4 **	69.4 ± 0.6	73.6 ± 0.5 **	70.1 ± 0.9	<0.001	72.9 ± 0.4	70.7 ± 0.5	74.2 ± 0.4 ***	71.8 ± 0.5	<0.001
Triglyceride (mg/dL)	120.6 ± 2.6 ***	120.9 ± 4.0 ***	152.2 ± 3.8	152.1 ± 7.5	<0.001	125.2 ± 3.0 ***	120.5 ± 3.0 ***	145.1 ± 5.3	153.8 ± 6.0	<0.001
Total cholesterol (mg/dL)	178.3 ± 1.5	175.9 ± 2.1	177.9 ± 1.8	177.2 ± 3.1	0.791	194.4 ± 1.5 ***	186.4 ± 1.9	189.3 ± 1.7	184.2 ± 2.1	<0.001
HDL-cholesterol (mg/dL)	48.0 ± 0.4 ***	45.8 ± 0.7 ***	44.9 ± 0.6 *	42.3 ± 0.9	<0.001	52.0 ± 0.5 ***	49.4 ± 0.7 ***	49.4 ± 0.5 ***	46.3 ± 0.5	<0.001
LDL-cholesterol (mg/dL)	111.5 ± 4.1	101.2 ± 6.3	103.0 ± 3.5	106.1 ± 6.1	0.316	108.5 ± 4.8	111.3 ± 4.9	107.3 ± 4.2	109.2 ± 3.6	0.944

^1^ Mean ± standard error. ^2^ *p*-value by general linear regression. * *p* < 0.05, ** *p* < 0.01, *** *p* < 0.001 versus sarcopenic obesity by LSD multiple comparisons.

**Table 3:** Health and dietary behavioral characteristics according to sarcopenia and obesity status by sex.

Variables	Men					Women				
	Normal	Sarcopenia	Obesity	Sarcopenic Obesity	*p*-Value ^2^	Normal	Sarcopenia	Obesity	Sarcopenic Obesity	*p*-Value
Smoking										
Current smoker	164 (16.8) ^1^	98 (20.9)	124 (17.3)	29 (12.5)	0.140	22 (2.1)	7 (1.6)	24 (2.1)	13 (3.3)	0.510
Past or non-smoker	841 (83.2)	357 (79.1)	576 (82.7)	172 (87.5)		961 (97.9)	559 (98.4)	899 (97.9)	532 (96.7)	
Alcohol consumption										
No	396 (38.3)	257 (56.0)	252 (35.5)	112 (54.4)	<0.001	779 (78.3)	484 (84.3)	752 (82.1)	469 (84.6)	0.034
Yes	609 (61.7)	200 (44.0)	448 (64.5)	91 (45.6)		204 (21.7)	86 (15.7)	171 (17.9)	77 (15.4)	
Stress										
Yes	98 (9.7)	69 (15.0)	77 (10.9)	29 (16.0)	0.028	219 (23.0)	151 (26.6)	189 (19.9)	144 (27.8)	0.022
No	906 (90.3)	383 (85.0)	623 (89.1)	172 (84.0)		764 (77.0)	415 (73.4)	733 (80.1)	400 (72.2)	
Nutrition education										
Yes	53 (5.8)	24 (3.8)	45 (6.2)	13 (8.3)	0.276	77 (6.9)	58 (8.8)	75 (7.5)	61 (11.0)	0.106
No	957 (94.2)	441 (96.2)	664 (93.8)	193 (91.7)		915 (93.1)	530 (91.2)	855 (92.5)	496 (89.0)	
Eating-out										
≥1 time/week	539 (55.4)	172 (37.7)	427 (63.1)	79 (41.8)	<0.001	449 (46.9)	190 (34.8)	389 (43.5)	217 (36.3)	<0.001
1–3 times/month	331 (31.4)	167 (35.1)	208 (27.9)	79 (35.4)		325 (31.7)	179 (29.3)	324 (32.7)	165 (29.1)	
Rarely (<1 time/week)	140 (13.2)	126 (27.2)	74 (9.0)	48 (22.8)		218 (21.4)	219 (35.9)	217 (23.8)	175 (34.6)	
Food security										
Food secure	226 (94.5)	91 (91.5)	210 (93.8)	55 (87.1)	0.383	251 (93.0)	109 (90.7)	285 (91.9)	112 (82.9)	0.024
Food insecure	14 (5.5)	6 (8.5)	16 (6.2)	7 (12.9)		17 (7.0)	14 (9.3)	27 (8.1)	24 (17.1)	
Dietary supplement										
Yes	497 (79.0)	175 (41.6)	366 (51.1)	88 (42.6)	0.035	598 (61.0)	329 (54.0)	505 (55.0)	283 (51.4)	0.016
No	513 (51.0)	290 (58.4)	343 (48.9)	118 (57.4)		394 (39.0)	259 (46.0)	425 (45.0)	274 (48.6)	
Physical activity										
>10 min (days/week)	3.1 ± 0.0 ^3,^*	2.7 ± 0.1	2.9 ± 0.1	2.8 ± 0.1	<0.001	3.0 ± 0.1 ***	2.4 ± 0.1	2.8 ± 0.1 ***	2.2 ± 0.1	<0.001
>30 min (days/week)	2.1 ± 0.1 **	1.6 ± 0.1	1.9 ± 0.1	1.7 ± 0.1	<0.001	1.9 ± 0.1 ***	1.4 ± 0.1 **	1.8 ± 0.1 ***	1.1 ± 0.1	<0.001
% Energy from meal										
Breakfast	26.2 ± 0.3	29.0 ± 0.5	26.2 ± 0.5	27.7 ± 0.7	<0.001	27.6 ± 0.5 ***	29.4 ± 0.5	27.9 ± 0.4 **	30.2 ± 0.6	<0.001
Lunch	30.4 ± 0.4	30.5 ± 0.6	32.2 ± 0.7	30.4 ± 1.1	0.106	31.0 ± 0.5	31.6 ± 0.7	32.1 ± 0.6	33.0 ± 0.8	0.180
Dinner	31.0 ± 0.4	30.2 ± 0.7	30.9 ± 0.5	31.7 ± 1.1	0.635	28.1 ± 0.4 **	29.5 ± 0.5	28.6 ± 0.5 *	30.4 ± 0.7	0.011
Snack	16.9 ± 0.5	16.7 ± 0.8	16.2 ± 0.7	16.9 ± 1.2	0.870	20.5 ± 0.6 **	17.9 ± 0.7	20.4 ± 0.7 **	17.3 ± 0.7	0.001

^1^ *n* (weighted %). ^2^ *p*-value by chi test for categorical variables and general linear regression for continuous variables. ^3^ Mean ± standard error. * *p* < 0.05, ** *p* < 0.01, *** *p* < 0.001 versus sarcopenic obesity by LSD multiple comparisons.

**Table 4:** Korean Healthy Eating Index (KHEI) score according to sarcopenia and obesity status by sex.

Variables	Men					Women				
	Normal	Sarcopenia	Obesity	Sarcopenic Obesity	*p*-Value ^2^	Normal	Sarcopenia	Obesity	Sarcopenic Obesity	*p*-Value
Adequacy										
Have breakfast (0–10)	9.6 ± 0.1 ^1^	9.7 ± 0.1	9.5 ± 0.1	9.7 ± 0.1	0.187	9.6 ± 0.1 **	9.5 ± 0.1 *	9.3 ± 0.1	9.0 ± 0.1	0.007
Mixed grains (0–5)	2.9 ± 0.1	2.3 ± 0.1 *	2.7 ± 0.1	2.8 ± 0.2	0.004	2.4 ± 0.1	2.3 ± 0.1	2.7 ± 0.1	2.4 ± 0.1	0.077
Total fruits (0–5)	2.6 ± 0.1 **	2.0 ± 0.1	2.5 ± 0.1	2.1 ± 0.2	<0.001	3.3 ± 0.1 ***	2.8 ± 0.1	3.2 ± 0.1 ***	2.5 ± 0.1	<0.001
Fresh fruits (0–5)	2.7 ± 0.1	2.2 ± 0.1	2.6 ± 0.1	2.4 ± 0.2	0.032	3.3 ± 0.1 ***	2.8 ± 0.1	3.2 ± 0.1 ***	2.6 ± 0.1	<0.001
Total vegetables (0–5)	3.9 ± 0.1 ***	3.4 ± 0.1	3.9 ± 0.1 ***	3.3 ± 0.1	<0.001	3.7 ± 0.1 **	3.3 ± 0.1	3.7 ± 0.1 **	3.4 ± 0.1	<0.001
Vegetables, excluding kimchi and pickles (0–5)	3.3 ± 0.1 ***	2.7 ± 0.1	3.4 ± 0.1 ***	2.8 ± 0.1	<0.001	3.6 ± 0.1 ***	3.1 ± 0.1	3.5 ± 0.1 *	3.2 ± 0.1	<0.001
Meat, fish, eggs and beans (0–10)	7.1 ± 0.1 **	5.6 ± 0.2 *	7.1 ± 0.2 *	6.3 ± 0.3	<0.001	7.1 ± 0.1 ***	5.8 ± 0.2	6.9 ± 0.1 ***	5.9 ± 0.2	<0.001
Milk and milk products (0–10)	2.4 ± 0.2	1.8 ± 0.2	2.4 ± 0.2	2.5 ± 0.3	0.058	3.0 ± 0.2 ***	2.9 ± 0.2 **	3.1 ± 0.2 ***	2.1 ± 0.2	<0.001
Moderation										
% Saturated fatty acid (0–10)	9.1 ± 0.1	9.2 ± 0.1	8.8 ± 0.1	9.0 ± 0.2	0.196	8.9 ± 0.1	9.1 ± 0.1	9.0 ± 0.1	9.0 ± 0.1	0.668
Sodium (0–10)	6.7 ± 0.1 **	7.8 ± 0.2	6.6 ± 0.2 **	7.5 ± 0.3	<0.001	8.5 ± 0.1	8.8 ± 0.1	8.2 ± 0.1	8.5 ± 0.1	0.007
% Sweets and beverage (0–10)	9.2 ± 0.1	9.2 ± 0.1	9.2 ± 0.1	9.1 ± 0.2	0.911	8.9 ± 0.1 ***	9.3 ± 0.1	9.1 ± 0.1 *	9.4 ± 0.1	0.002
Balance of energy intake										
Carbohydrate (0–5)	2.0 ± 0.1	1.5 ± 0.1	2.1 ± 0.1	1.7 ± 0.2	<0.001	1.7 ± 0.1 *	1.3 ± 0.1	1.7 ± 0.1 **	1.3 ± 0.1	<0.001
Fat (0–5)	2.9 ± 0.1 *	2.3 ± 0.1	3.0 ± 0.1 **	2.4 ± 0.2	<0.001	2.6 ± 0.1 **	2.2 ± 0.1	2.5 ± 0.1 **	2.1 ± 0.1	0.003
Total energy (0–5)	3.4 ± 0.1	3.1 ± 0.1	3.1 ± 0.1	3.1 ± 0.2	0.027	3.1 ± 0.1 *	2.8 ± 0.1	3.2 ± 0.1 *	2.9 ± 0.1	0.009
Total KHEI score (0–100)	67.9 ± 0.5 **	62.7 ± 0.7	66.7 ± 0.6	64.7 ± 0.9	<0.001	69.7 ± 0.5 ***	66.0 ± 0.7	69.3 ± 0.5 ***	64.4 ± 0.7	<0.001

^1^ Mean ± standard error. ^2^ *p*-value by general linear regression. * *p* < 0.05, ** *p* < 0.01, *** *p* < 0.001 versus sarcopenic obesity by LSD multiple comparisons.

**Table 5:** Nutrient intake according to sarcopenia and obesity status by sex.

Nutrients	Men					Women				
	Normal	Sarcopenia	Obesity	Sarcopenic Obesity	*p*-Value ^2^	Normal	Sarcopenia	Obesity	Sarcopenic Obesity	*p*-Value
Energy (kcal)	1963.4 ± 25.3 ^1,^**	1724.4 ± 34.6	1981.3 ± 42.0 *	1795.7 ± 57.1	<0.001	1474.9 ± 25.9 *	1391.8 ± 28.0	1477.3 ± 24.8 *	1393.8 ± 29.4	0.027
Carbohydrate (g)	321.3 ± 2.2	316.5 ± 3.9	311.5 ± 2.7	312.3 ± 5.2	0.059	256.7 ± 1.8	261.2 ± 1.8	257.7 ± 1.8	260.5 ± 2.4	0.316
Protein (g)	64.6 ± 0.6	63.2 ± 1.1	65.6 ± 0.8	65.8 ± 1.3	0.203	47.6 ± 0.5	45.6 ± 0.6	47.4 ± 0.6	46.9 ± 0.7	0.082
Fat (g)	30.7 ± 0.6	30.7 ± 0.9	33.3 ± 0.8	32.6 ± 1.7	0.062	24.2 ± 0.6	22.8 ± 0.7	23.8 ± 0.6	22.5 ± 0.8	0.320
Dietary fiber (g)	29.1 ± 0.5 ***	27.0 ± 0.8	28.3 ± 0.5 **	25.7 ± 0.8	0.002	23.4 ± 0.4	22.4 ± 0.5	24.5 ± 0.6	23.0 ± 0.6	0.023
Calcium (mg)	522.4 ± 12.0	480.3 ± 17.1	507.1 ± 13.4	494.6 ± 20.2	0.226	408.9 ± 10.8	396.3 ± 10.5	381.5 ± 7.5	379.9 ± 10.1	0.093
Phosphorus (mg)	1055.7 ± 10.5	1013.2 ± 16.5	1049.2 ± 12.2	1030.6 ± 18.8	0.126	798.0 ± 8.5	771.2 ± 8.5	786.0 ± 8.9	767.3 ± 10.8	0.089
Sodium (mg)	3396.1 ± 67.9	3256.5 ± 93.6	3400.0 ± 71.1	3186.2 ± 126.1	0.317	2220.7 ± 47.9 **	2365.5 ± 79.6	2448.1 ± 58.2	2494.6 ± 85.4	0.008
Potassium (mg)	2938.2 ± 38.1 *	2789.5 ± 55.0	2919.4 ± 46.2 *	2759.4 ± 65.8	0.027	2333.4 ± 36.3	2253.0 ± 35.6	2411.3 ± 41.1 **	2245.9 ± 43.8	0.008
Iron (mg)	12.6 ± 0.2	12.3 ± 0.5	12.2 ± 0.2	11.7 ± 0.4	0.113	9.5 ± 0.2	9.3 ± 0.2	9.6 ± 0.2	9.3 ± 0.2	0.581
Vitamin A (μg RAE)	326.3 ± 11.9	320.9 ± 21.8	382.1 ± 35.6	374.0 ± 27.2	0.134	268.3 ± 9.7	271.0 ± 11.4	267.2 ± 9.2	262.8 ± 15.2	0.966
Thiamine (mg)	1.30 ± 0.02	1.30 ± 0.03	1.29 ± 0.03	1.36 ± 0.04	0.481	0.97 ± 0.01	1.00 ± 0.02	0.99 ± 0.01	0.99 ± 0.02	0.587
Riboflavin (mg)	1.38 ± 0.02	1.24 ± 0.03	1.39 ± 0.03	1.32 ± 0.04	<0.001	1.05 ± 0.02	0.99 ± 0.02	1.03 ± 0.02	1.00 ± 0.03	0.159
Niacin (mg)	12.3 ± 0.2	11.8 ± 0.2	12.3 ± 0.2	12.3 ± 0.4	0.288	8.8 ± 0.1	8.7 ± 0.2	8.9 ± 0.1	8.7 ± 0.2	0.772
Vitamin C (mg)	61.0 ± 2.4	57.9 ± 3.8	57.4 ± 3.3	51.6 ± 3.1	0.158	56.6 ± 2.6 **	48.8 ± 2.3	56.6 ± 2.8 *	47.7 ± 2.3	0.017
% Energy from										
Carbohydrate (%)	68.3 ± 0.4	67.9 ± 0.8	66.9 ± 0.5	67.2 ± 1.0	0.190	71.6 ± 0.5	73.2 ± 0.5	72.0 ± 0.5	72.6 ± 0.6	0.134
Protein (%)	13.5 ± 0.1	13.1 ± 0.2	13.7 ± 0.2	13.9 ± 0.3	0.139	13.1 ± 0.1	12.5 ± 0.1 *	13.1 ± 0.2	13.0 ± 0.2	0.022
Fat (%)	14.1 ± 0.3	13.9 ± 0.5	14.9 ± 0.3	14.5 ± 0.7	0.173	14.5 ± 0.3	13.5 ± 0.4	14.3 ± 0.3	13.4 ± 0.5	0.138

^1^ Mean ± standard error. ^2^ Means and *p*-value by generalized linear regression adjusted for age and energy intake. * *p* < 0.05, ** *p* < 0.01, *** *p* < 0.001 versus sarcopenic obesity by LSD multiple comparisons.

**Table 6:** Odd ratios for sarcopenia and obesity status according to KHEI scores by sex.

		Men			Women		
		Model 1	Model 2	Model 3	Model 1	Model 2	Model 3
Total KHEI score							
Sarcopenia	Q4 (ref.)	1	1	1	1	1	1
	Q3	1.58 (1.02–2.43) ^1,^*	1.37 (0.87–2.16)	1.43 (0.86–2.38)	1.28 (0.90–1.82)	1.04 (0.71–1.52)	1.04 (0.67–1.60)
	Q2	2.15 (1.44–3.22) ***	1.20 (1.32–3.02) ***	2.06 (1.30–3.28) **	1.51 (1.05–2.19) **	1.01 (0.69–1.47)	0.99 (0.65–1.54)
	Q1	3.46 (2.27–5.26) ***	3.02 (1.96–4.66) ***	2.76 (1.67–4.58) ***	2.31 (1.60–3.34) ***	1.27 (0.86–1.87)	1.12 (0.70–1.78)
Obesity	Q4 (ref.)	1	1	1	1	1	1
	Q3	1.31 (0.97–1.79) ^1^	1.31 (0.96–1.78)	1.45 (1.03–2.05)	1.18 (0.87–1.59)	1.15 (0.85–1.56)	1.11 (0.79–1.57)
	Q2	1.26 (0.90–1.78)	1.27 (0.90–1.79)	1.26 (0.87–1.84)	1.24 (0.91–1.70)	1.18 (0.86–1.61)	1.14 (0.81–1.61)
	Q1	1.62 (1.13–2.32) **	1.61 (1.12–2.30) *	1.70 (1.14–2.56) *	1.13–0.81–1.58)	1.04 (0.75–1.46)	0.89 (0.61–1.29)
Sarcopenic obesity	Q4 (ref.)	1	1	1	1	1	1
	Q3	1.22 (0.71–2.09) ^1^	1.09 (0.61–1.96)	0.91 (0.45–1.85)	1.54 (1.10–2.15) *	1.30 (0.93–1.84)	1.09 (0.71–1.67)
	Q2	1.80 (1.11–2.91) *	1.59 (0.97–2.60)	1.20 (0.69–2.08)	2.01 (1.41–2.87) ***	1.52 (1.05–2.20) *	1.33 (0.86–2.04)
	Q1	2.00 (1.22–3.27) **	1.75 (1.05–2.91) *	0.98 (0.55–1.78)	2.92 (2.01–4.23) ***	1.70 (1.13–2.54) *	1.22 (0.74–2.01)
Meat, fish, eggs, and beans intake							
Sarcopenia	Q4 (ref.)	1	1	1	1	1	1
	Q3	0.96 (0.59–1.54)	0.88 (0.54–1.44)	0.90 (0.51–1.59)	1.77 (1.22–2.56) *	1.61 (1.06–2.42)	1.58 (1.01–2.48) *
	Q2	2.38 (1.65–3.42) ***	2.09 (1.42–3.08) **	1.81 (1.14–2.87) *	1.90 (1.34–2.69) *	1.39 (0.98–1.99)	1.34 (0.91–1.98)
	Q1	2.52 (1.75–3.64) ***	1.92 (1.30–2.86) **	1.77 (1.13–2.78) *	2.76 (1.96–3.88) ***	1.63 (1.15–2.32) **	1.45 (0.96–2.19)
Obesity	Q4 (ref.)	1	1	1	1	1	1
	Q3	0.83 (0.57–1.20)	0.82 (0.57–1.19)	0.74 (0.49–1.13)	1.08 (0.76–1.51)	1.07 (0.76–1.50)	1.06 (0.71–1.59)
	Q2	0.97 (0.72–1.31)	0.96 (0.71–1.30)	0.87 (0.62–1.21)	1.01 (0.75–1.37)	0.97 (0.72–1.31)	0.86 (0.61–1.21)
	Q1	0.96 (0.68–1.34)	0.93 (0.66–1.31)	0.91 (0.62–1.34)	1.23 (0.90–1.68)	1.15 (0.83–1.57)	0.99 (0.69–1.43)
Sarcopenic obesity	Q4 (ref.)	1	1	1	1	1	1
	Q3	1.32 (0.77–2.26)	1.20 (0.67–2.12)	0.99 (0.52–1.90)	1.25 (0.84–1.88)	1.18 (0.78–1.77)	1.10 (0.68–1.78)
	Q2	2.07 (1.26–3.40) *	1.78 (1.06–2.98) *	1.27 (0.72–2.27)	1.38 (0.99–1.92) **	1.17 (0.83–1.65)	1.02 (0.68–1.52)
	Q1	1.70 (1.02–2.84) ***	1.25 (0.73–2.16)	1.07 (0.59–1.91)	2.36 (1.62–3.45) ***	1.72 (1.14–2.58) **	1.28 (0.79–2.07)

^1^ Odds ratio (95% confidence interval) by multiple logistic regression. Model 1: Unadjusted. Model 2: Adjusted for age. Model 3: Adjusted for age, education, household income, and physical activity for more than 30 min (days/week) in men and for age, education, region, household income, and physical activity for more than 30 min (days/week) in women. The odds ratios can be interpreted as the risk for sarcopenia, obesity, and sarcopenic obesity relative to the normal status. * *p* < 0.05, ** *p* < 0.01, *** *p* < 0.001.

## Data Availability

The KNHANES datasets used in the current study can be obtained from following link: https://knhanes.kdca.go.kr/knhanes/sub03/sub03_02_05.do (accessed on 15 December 2023).

## Supplementary Materials

The following supporting information can be downloaded at: https://www.mdpi.com/article/10.3390/nu16081175/s1, Table S1: Korean Healthy Eating Index components and standards for scoring. Reference [38] is cited in Supplementary Materials.
